# Institutional Questions

**Published:** 1900-01-27

**Authors:** 


### INSTITUTIONAL QUESTIONS.

#### Hospital Account Keeping.

"Matron" asks: Should a hospital auditor have laid before him receipted bills, or only the slips which many firms fix on the bills to show the accounts are paid? The secretary's office here hold the bills which have been passed by the committee should be treated as sacred documents, and not allowed out of our custody, although not initialled by the chairman. Hence it is impossible for me to get the actual bills receipted in such cases when the accounts must be settled by post, and there is nothing beyond the similarity of the sums on the bill and separate receipt to show any connexion between them.

*** It is usual for tradesmen to send an invoice with all goods supplied, such invoice being produced to the Finance Commiteee and duly posted in the day-book by the accountant. It is usual for tradesmen to render duplicate copies of all bills when asked to do so, a practice which disposes of the difficulty which "Matron" raises. We are glad to note that the secretary holds his committee in such awe as to regard any document which may have been submitted to them as "sacred."

